# Risk Prediction by Using Artificial Neural Network in Global Software Development

**DOI:** 10.1155/2021/2922728

**Published:** 2021-12-09

**Authors:** Asim Iftikhar, Muhammad Alam, Rizwan Ahmed, Shahrulniza Musa, Mazliham Mohd Su'ud

**Affiliations:** ^1^College of Computer Science and Information Systems, Institute of Business Management (IoBM), Korangi Creek, Karachi, Pakistan; ^2^Malaysian Institute of Information Technology, Universiti Kuala Lumpur (UniKL MIIT), Kuala Lumpur, Malaysia; ^3^Multimedia University (MMU), Cyberjaya, Malaysia; ^4^Riphah Institute of System Engineering (RISE), Faculty of Computing, Riphah International University, Islamabad, Pakistan; ^5^Malaysian France Institute, Universiti Kuala Lumpur (UniKL MFI), Kuala Lumpur, Malaysia

## Abstract

The demand for global software development is growing. The nonavailability of software experts at one place or a country is the reason for the increase in the scope of global software development. Software developers who are located in different parts of the world with diversified skills necessary for a successful completion of a project play a critical role in the field of software development. Using the skills and expertise of software developers around the world, one could get any component developed or any IT-related issue resolved. The best software skills and tools are dispersed across the globe, but to integrate these skills and tools together and make them work for solving real world problems is a challenging task. The discipline of risk management gives the alternative strategies to manage risks that the software experts are facing in today's world of competitiveness. This research is an effort to predict risks related to time, cost, and resources those are faced by distributed teams in global software development environment. To examine the relative effect of these factors, in this research, neural network approaches like Levenberg–Marquardt, Bayesian Regularization, and Scaled Conjugate Gradient have been implemented to predict the responses of risks related to project time, cost, and resources involved in global software development. Comparative analysis of these three algorithms is also performed to determine the highest accuracy algorithms. The findings of this study proved that Bayesian Regularization performed very well in terms of the MSE (validation) criterion as compared with the Levenberg–Marquardt and Scaled Conjugate Gradient approaches.

## 1. Introduction

The last two decades have changed the world [1]. Looking at the past where you had to walk to meet your relative, let aside talking to someone face to face at the other end of the world. The idea of global village is not a direct result of having an excessive number of restaurants or high rise structures but it is because of the use of advanced technology for an effective and efficient exchange of information throughout the world. Similarly, the field of software development has also experienced the effect of the rapidly changing world to adapt as per the needs of their clients. In order to offer advantages over conventional techniques used in software development, the localized environment has transformed to the distributed environment [2]. In the last decade, many software firms began to discover or test the distributed software development facilities and subcontracted the projects in search of cheaper and skilled resources as an alternative [3]. As a result, software development has become a multisite, diverse, and globally a distributed work. At various levels, the designers, engineers, managers, and officials involved in software development have been facing the challenges of social and cultural diversity in accomplishing the task [3, 4].

Due to the technological advancement, the field of software development is growing at a very fast pace both locally and globally and it has been now referred as global software development (GSD), offshore software development, or development by outsourcing. Software outsourcing is a corporate level strategy that has been adopted for the past two decades and is gaining maturity [5]. The software outsourcing is a GSD outsourcing model for producing high-quality software at lower costs [6, 7]. Despite of the increasing growth, the software development industry has some entry barriers as the industry lacks a defined model of execution [8]. Globalization, in simple terms means ‘one world connected together.' However, technology does not understand humans. Humans are different by nature which essentially means it is absolute to say the term globalization implies cultural heterogeneity [9, 10]. Today, businesses, companies, and NGOs are investing its financial capital to develop and understand human dynamics. Companies are currently involved in enhancing its cross-cultural understanding and inculcating intercultural communication skills and intensifying management competencies. All of this requires great deal of time and money, but companies are willing to do all this because the organizations that take the cultural context of their teams into account generally experience greater project success [11].

**Figure 1:**
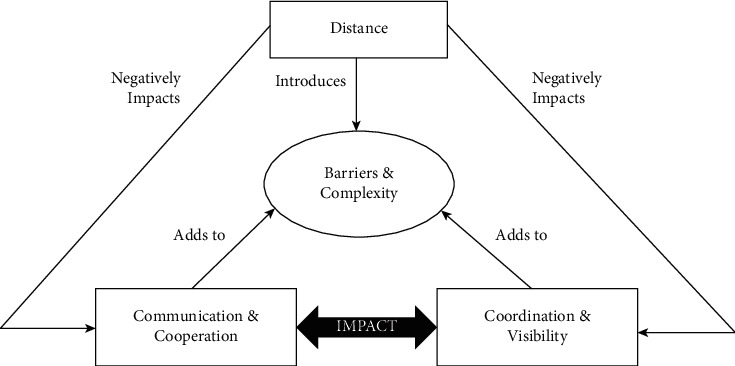
Issues in global software development [16].

GSD is a model of the modern age. Software team members are dispersed as they operate across different geographical locations, at different time zones and organizational boundaries, communicate concurrently using tools, and exchange information irrespective of time difference and physical boundaries [12]. Among the entire IT organization and industry, GSD is very popular. A large number of employees from this industry work on global projects because of the benefits that it offers. It is very time consuming but is very beneficial. The advantages of GSD include global development, cheap and skilled labor, better product quality, round the clock development, work efficiency, economic benefit, and many more [2, 13, 14]. Besides all these key factors and benefits, labor working in the GSD environment faces many difficulties and challenges as well, such as strategic issues, lack of communication or improper communication, different sociocultural backgrounds, and project management may also be one of the main concerns [4, 15]. The schematic diagram of issues associated with GSD is shown in Figure 1 [16].

GSD projects can be divided as offshore and onshore. Offshore projects are not considered much successful. This is because of the physical time constraints, knowledge level, and cultural differences. Lack of communication also hampers the process of knowledge and data sharing between the two teams working at offshore and onshore locations [17]. This results in less productivity and quality and relatively consumes more time [18, 19]. It is suggested that the project manager before taking charge of any project that involves several countries or different distributed areas must inform about all these difficulties and operational risk that they may face [18–21].

Risk can also be defined as the possibility of an event occurring that may end up having either a negative or a positive bearing on the overall objective [22]. One of the critical functions of management strategies is risk management. It is the internal control mechanism driven with certain set of designed practices and procedures in order to properly manage the loopholes within the system. Moreover, it also includes identifying, analyzing, handling, evaluating, inspecting, and reviewing risk [23, 24].

In project management, risk management plays a vital role in preventing and mitigating risks that have the potential to adversely affect the desired outcomes. In small- and medium-sized enterprises (SMEs), preventive measures are incorporated to minimize risks such as insurance and creation of reserves as part of the risk management process [25]. All SMEs need a well-planned risk management strategy to combat any adverse effect of an unexpected problem during the project [26].

Besides the benefits and risks, the threats are also involved while managing the GSD projects. As the team is located in several countries or designated in different regions, there may be obstacles like geographical risk, language barrier, political concerns, and weather issues that are to be considered while working on a global software development project. [27].

In this research, the techniques of artificial neural network (ANN)-based training algorithms, Levenberg–Marquardt (LM), Bayesian Regularization (BR), and Scaled Conjugate Gradient (SCG) have been implemented to predict the risks involved in GSD environment. Results are presented using performance plot, training state plot, error histogram, and regression plot. To identify the best performance, comparative analysis of three implemented algorithms has also been performed.

Most of the software organization faces risks in GSD environment. They mitigate the risk using traditional risk management tools. In addition, they realize that the traditional approaches of risk management lack the ability to address crucial characteristics of GSD in any significant detail. This is why the utilization of machine learning (ML) techniques to manage risk is helpful due to the self-learning and self-healing nature of ML algorithms. It becomes handy because such ML algorithms can deal with unstructured information. This research shows that ML provides effective techniques that help to predict the risks associated with GSD. It has a great contribution to optimize the process of GSD to make it competitive and efficient in terms of fulfilling the needs of the firms that depends upon IT infrastructure. Such tools can assist project managers in decision making in such a way that they can predict future risks with respect to project time, cost, and resource needed in the completion of the project.

This paper comprises of five (5) sections. The first section covers the introduction of this research study. Related work with respect to research has been explained in Section 2. The artificial neural network algorithm and its techniques utilized in this research are discussed in Section 3. Research methodology is elaborated in Section 4.  Section 5 discusses the results and findings, and the last section concludes this research.

## 2. Related Work

In [28], authors established a multilayer feed forward backpropagation-based neural network by utilising seven defect sets of data from the PROMISE repository. Levenberg–Marquardt (LM), Bayesian Regularization (BR), and Resilient backpropagation (RP) training algorithms were implemented with the criteria of statistical estimations like MSE and R2 results, and the boundaries were calculated from the confusion matrix. BR training techniques were optimal as compared with the LM and RP methods to decrease the mean square error and type 2 error that consequently increased accuracy, sensitivity, and R2 values. Precision of greater than 90% was yielded by BR on each of the seven datasets.

The degree of requirement complexity was utilized as a predictor of software complexity by the authors in [29]. The data pattern makes it difficult to draw a connection between a need and its complexity. To that end, this article attempts to create a connection model that connects the complexity of software requirements to software complexity predictions utilising the artificial neural network technique and the Levenberg–Marquardt and Bayesian Regulation algorithms. Conclusion presents that the Levenberg–Marquardt method is superior at predicting software complexity based on complexity requirements since the resulting mean square error is lower.

Fuzzy logic (FL) and neural Nnetwork (NN) methods were used by researchers in [30] to forecast software dependability. This article makes use of four different techniques to estimate the dependability of the dataset that was obtained from John Musa at Bell Labs. A neural network is a hybrid of a neural network and a fuzzy network. After analyzing the data, the fuzzy-neural approach produced the best results out of all the ones that were considered. The Levenberg–Marquardt algorithm is used to train the neurons in the fuzzy-neural approach. Our suggested methods have been put to the test utilising testing data, which represents 15% of the failure data set's total data.

For defect prediction in software, three cost sensitive enhanced algorithms have been studied by researchers in [31] to enhance the neural network. Transferring the organizing threshold towards not-fault-prone modules had been organized properly by using the first algorithm that depends on threshold moving. Also, to boost the additional weights on the sample that are aligned with unclassified defect-prone modules, the rest of the two weight-updating-based algorithms integrated the misdeem price into the weight-update rule. The normalized expected cost of misclassification (NECM) identified by measuring the performance of all the above algorithms had been assessed with the help of four datasets from NASA-based projects. From the experimental results, it had been observed that threshold moving found as the best option to make the software that is more sensitive in terms of cost. It had also predicted the defect in software with boosted neural networks among all the algorithms considered exclusively those type of datasets that were developed with the help of object-oriented language.

The chronic kidney disease (CKD) is one such disease for which in [32] researchers have developed a detection system using the artificial neural network. The idea here is to use intelligent systems to calculate the probability of having a particular illness in otherwise normal people. They used UCI ML repository's input data for training, validation, and testing, and they found that the Levenberg–Marquardt is the best on efficiency as compared with Levenberg and Bayesian regularization. They are reporting 99.8% accuracy with the Levenberg–Marquardt algorithm. This is an excellent example of using neural networks to find an economical solution to CKD detection.

Researchers in [33] used the Levenberg–Marquardt, Bayesian Regularization, and Scaled Conjugate Gradient learning algorithms to predict the survival of a diabetes patient as an exercise in medical diagnosis. They discussed the performance of these supervised machine learning algorithms through regression analysis as they used Diabetes Dataset of the Pima Indian living in Arizona, theUSA, for training and testing the network. They also found that the Levenberg–Marquardt algorithm is the most suitable algorithm for prediction.

The cryptocurrency and digital currencies are growing fast as an alternative to the fiat currency. Authors in [34] used the feedforward neural network (FNN) to predict the behavior of Bitcoin which is highly popular decentralized cryptocurrency in contemporary times. The Bitcoin price characteristics are nonstationary and nonlinear which according to the authors makes the neural network techniques more suitable to study their behavior in time as the classical models cannot handle nonlinearity. The researchers found that the usage of the Levenberg–Marquardt backpropagation algorithm (FNN-LM) worked much better to forecast the price of Bitcoin as compared with the Scaled Conjugate Gradient (SCG) backpropagation algorithm. The better performance of FNN-LM is interesting to note because one would assume that the SCG algorithm will work better in such a case of large network and dataset.

Due to improvement in communication, the world has become a global village resulting in much of the software development taking place between groups situated in different countries. The measuring of software complexity is important because a code which is considered at a level of “good-complexity” is easier to understand, faster to debug, comfortable to maintain, and contains less errors. What is a “good-complexity” level? To answer this question, different metrics of software complexity are in use which include line of code metric, Halstead metric, and maintainability index to mention a few. The researchers in [35] proved that neural networks are fairly accurate for calculating software complexity metric. They argue in their paper that the results from Bayesian Regularization algorithm show average difference of only 0.09% for Volume, 1.08% for Effort, and 0.36% for the Program Length as compared with the Halstead metric. This shows that neural networks are an effective tool for estimation of software complexity.

The researchers in [36] used neural network algorithms for predicting physical properties of superconductors and concluded that Levenberg–Marquardt provides the best performance as it gives a fairly accurate prediction for the critical temperature of superconductors which the authors did through plotting SOM (Self Organizing Maps) derived from applying to data set through neuro fuzzy networks. Neural network algorithms may be of great help (i) to narrow down the right materials parameters to work with to prepare new materials in the laboratories and (ii) to accurately predict the critical temperature [37].

## 3. Problem Statement

Software experts in global software development environment are facing many challenges. Moreover, risk is a big challenge as compared with other challenges. In GSD, where team members work in different geographical locations and different time zones, the risks related to project time, cost, and resources should be taken into account so that project managers can take better decisions to reduce these risks. This research article is an effort to focus on the implementation of neural network approaches (Levenberg–Marquardt, Bayesian Regularization, and Scaled Conjugate Gradient) to predict overall project risks according to time, cost, and resources which will help decision makers to assess time, budget, and resources needed to conduct a project.

## 4. Artificial Neural Network

**Figure 2:**
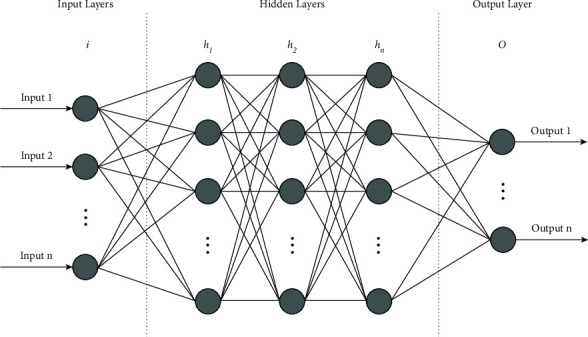
Artificial neural network architecture [38].

Artificial neural networks (ANNs) are models inspired by the biological networks, especially the neural network in the human or animal brain. It tries to imitate functions of the human brain like speech recognition, pattern recognition, and face recognition which are just a few of the neurological processes that the human brain performs [38]. The network learns from examples and continues the iteration process with forward and backward propagation. This process is done until the output is in conformity with the provided response with certain desired accuracy. Every example consists of series of inputs and corresponding outputs also known as responses, and the network makes changes through the internal connections known as weights [39]. A typical ANN consists of three layers (see Figure 2 [40]): (1) an input layer, (2) a hidden layer, and (3) an output layer. For example, an input layer could be an object (like a cube), and the output goal is to recognize this cube and identify it accurately. The task of recognizing the cube is performed with the help of hidden layer through an iteration process. The input layer, the hidden layer, and the output layer are connected using connections (or channels) as shown by solid lines.

The three approaches pertinent to the artificial neural network algorithm named as Levenberg–Marquardt, Bayesian Regularization, and Scaled Conjugate Gradient training algorithms had been implemented in various research papers to examine to relative efficiency of these three approaches in terms of predictive ability.

### 4.1. Levenberg–Marquardt

It is an algorithm that trains ANN relatively quicker than the backpropagation algorithm. In this approach, the minimum of a function is expressed as a sum of squares of a nonlinear function. [41]. The Levenberg–Marquardt method implements the least damped square method with respect to weights [42–44]. The Levenberg–Marquardt backpropagation algorithm uses the conjugate gradient technique to reduce the sum of squares at each iteration [45]. It was intended not to calculate the Hessian matrix [46].

### 4.2. Scaled Conjugate Gradient

The Scaled Conjugate Gradient algorithm belongs to the group of Conjugate Gradient methods [41]. It starts initially in the direction with steepest descent that allows it to converge to the minimum error [42]. This method avoids a line-search per learning literature by using a step size scaling mechanism, and it makes this algorithm faster as compared with other second-order algorithms. Scaled Conjugate Gradient offers a controlled learning algorithm with a super linear convergence rate [43]. The Scale Conjugate method is comparatively faster than the standard backpropagation as it does not include any critical user-dependent parameter. It can train any network as long as its weight, net input, and transfer functions have derivative functions [44].

This algorithm was built on conjugate directions and not on a line-search. The stopping criteria are either a maximum number of epochs or the maximum amount of time exceeds or the performance reaches below to minimum gradient [46, 47].

### 4.3. Bayesian Regularization

As compared with the standard backpropagation method, Bayesian Regularization is relatively more robust which eliminates the need for cross-validation [44]. It provides a probability distribution to be used in quantitative reasoning analysis. The Bayesian regularization algorithm contains an objective function that includes a residual sum of squares and sum of squared weights to minimize estimation errors for obtaining the required model [45, 46].

It belongs to the group of probabilistic graphical simulations based on a set of random variables and directed acyclic graphs to exhibit the probable dependence between variables [48].

Several researchers have used these three ANN techniques with a variety of applications. In [49], authors applied these three ANN techniques for the prediction of Flash Floods and found Bayesian Regularization an efficient technique as compared with the other two techniques. Authors in [50] used ANN for reservoir petro-physical properties such as porosity, permeability, and water saturation. In [51], authors compared relative predictive abilities of Levenberg–Marquardt and Bayesian Regularization methods for data on prices of four cryptocurrencies, namely, Bitcoin, Bitcoin Cash, Litecoin, and Ripple and found that the Bayesian Regularization method gives less error in prediction as compared with Levenberg–Marquardt in case of large data but both the methods are found nearly equally efficient for small data. Researchers in [52] used the Bayesian Regularization method to predict stock time series and worked for the improvement of the Bayesian Regularization method to increase the predictive ability of time series data used in the study. Authors in [53] applied four backpropagation neural networks to explore correlation that how plug load data, occupancy rates, and local weather factors affects to predict electricity usage. Authors in [54] studied the challenges to reduce complexity, failures, and time in the field of software development and used fuzzy-neural network composed by fuzzy rules to seek the solutions to overcome these challenges.

## 5. Research Methodology

Under umbrella of the artificial neural network algorithm, Levenberg–Marquardt, Bayesian Regularization, and Scaled Conjugate Gradient training algorithms have been implemented for risk prediction in global software development projects.

### 5.1. Research Design

This study utilized both experimental and simulation-based research designs. Using convenient sampling, a total of 274 medium- and large-sized software organizations in Pakistan, Australia, and the United States have been identified from where data can be collected. However, first, a thorough study of the available literature has been carried out to identify risks associated with project time, cost, and resources.

A total of 54 risks related to project time, cost, and resource were identified from literature review that would have been sufficient to compromise the effectiveness and viability of a software project in software development. List of 54 identified risk factors was sent to industry connoisseurs; the industry experts decreased the 54 risk factors to 26 (see Table 1) that could affect negatively the project in GSD environment. A questionnaire was designed to address the 26 risk factors emphasized by the software experts. The questionnaire was sent to 760 medium- and large-sized software development firms in Pakistan, Australia, and the USA. Data cleaning has been done because some organizations left certain questions incomplete.

There are mainly three types of risk factors that are cumulatively linked to an overall risk in a project related to GSD, i.e., time risk, cost risk, and resource risk. However, the extent of the risk posed by each risk factor may differ in weightage or effect on an overall risk. Therefore, a thorough examination of the three risks is needed to find the extent to which each risk contributes to an overall risk in the project. To address this, three neural network approaches named as Levenberg–Marquardt (LM), Bayesian Regularization (BR), and Scaled Conjugate Gradient (SCG) have been implemented to predict these risks in GSD.

**Figure 3:**
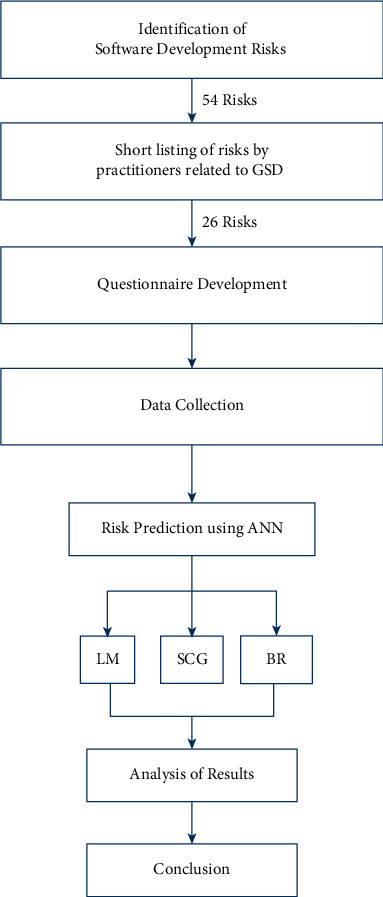
Research framework.

Finally, results have been achieved, and comparative analysis of LM, BR, and SCG has been performed with respect to project time, cost, and resource-related risks (see Figure 3).

### 5.2. Respondents of the Study

The people who replied to the questionnaire were the project managers, team leaders, and system and business analysts from medium- to large-sized software development organizations, located in Pakistan, Australia, and the United States. They are the people who have to face different types of risk in GSD environment. A total of 107, 103, and 64 responses were received from the American, Australian, and Pakistani software organizations.

### 5.3. Data Collection Procedure

The risks concerning the challenges of global software development were investigated using the questionnaire survey method. The questionnaire contained 33 questions related to time, cost, and resource risks. Out of these 33 questions, Q13, Q14, Q15, Q17, Q19, Q20, Q26, Q27, and Q28 covered the risk related to time; Q8, Q13, Q14, Q15, Q18, Q26, Q27, and Q28 encompass risk pertinent to cost whereas Q8, Q10, Q11, Q15, Q16, Q21, Q22, Q23, Q24, and Q25 to risk caused pertinent to resource (for ouestionnaire see Appendix).

The respondents were given the options as follows: 0 (Very Unlikely), 1 (Unlikely), 2 (Neutral), 3 (Likely), and 4 (Very Likely).

The questionnaire was sent to 760 medium- and large-sized software development organizations located in Pakistan, Australia, and the United States. A total of 390 responses were received; out of which, 116 responses were rejected considered as invalid. The sample data of 274 respondents are given in Tables 2 and 3. These data have been trained using neural network approaches like Levenberg–Marquardt, Bayesian Regularization, and Scaled Conjugate Gradient to perform data analysis.

## 6. Results and Findings

LM, BR, and SCG neural network training algorithms have been implemented, and mean squared error (MSE), regression *R* values, processing time, performance and gradient are calculated (see Tables 3–5). Performance plot, training state plot, error histogram, and regression plot have also been shown. Mean squared error (MSE), shown in equation (1), identifies the extent of the difference between the actual and the estimated values of the dependent variable, the lower the MSE value, the higher the goodness of fit and vice versa. The coefficient of correlation (R), as shown in equation (2), indicates the extent to which the fitted model is able to explain variation in the dependent variable due to the variation in the independent variable. The error histogram explains whether the errors are normally distributed which is critical for the goodness of fit of the model. Time-related risk is used as a predictor of overall risk associated to the global software development. The results of three different approaches LM, SCG, and BR are compared using the criterion of MSE and *R* to identify the best model.

(1) $$\begin{array}{c} \text{MSE}=\frac{1}{n}\sum_{i=1}^{n}{\left(y_{i}-{\hat{y}}_{i}\right)}^{2}, \end{array}$$

(2) $$\begin{array}{c} R=\frac{n\left(\sum ij\right)-\left(\sum i\right)\left(\sum j\right)}{\sqrt{\left[n\sum i^{2}-{\left(\sum i\right)}^{2}\right]\left[n\sum j^{2}-{\left(\sum j\right)}^{2}\right]}}. \end{array}$$

**Figure 4:**
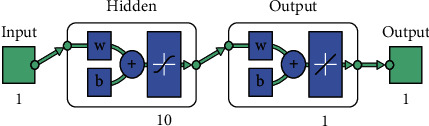
Neural network training.

LM, BR, and SCG-based neural networks are trained in which 1 input layer, 1 hidden layer, and 1 output layer are used (see Figure 4).

To predict the effect of time-related risk on the overall risk pertinent to global software development, three approaches of neural networks have been used. The results summarized (see Table 4) reveal that the MSE (validation) of the Bayesian Regularization method is the least with the maximum number of iterations for Epoch whereas MSE (testing) value of Levenberg–Marquardt is least with almost similar value of *R* as compared with the results for the same indicators of the other two models. Also, performance has the highest value in case of the Levenberg–Marquardt method. Epoch is computed with the least number of iterations in case of Levenberg–Marquardt as compared with the number of iterations associated to the other two approaches.

**Figure 5:**
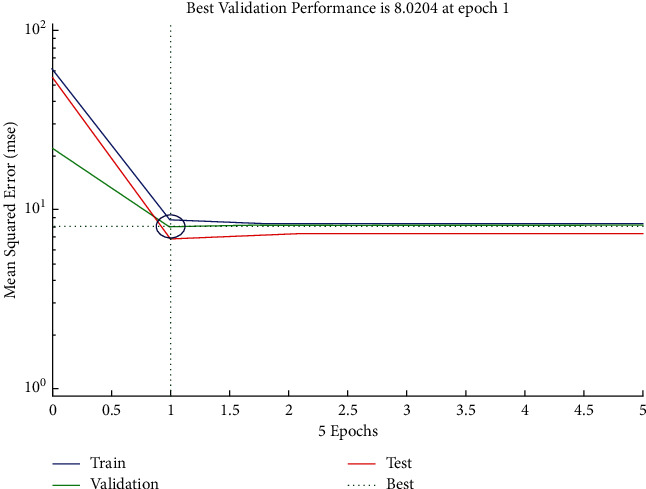
LM performance plot of time-related risk.

The performance plot of time-related risk in which 5 Epoch values are plotted versus mean squared error using Levenberg–Marquardt approach is shown in Figure 5. The plot shows the decreasing behavior between 0 and 1 and then follows the horizontal line trend for all values greater than 1.

**Figure 6:**
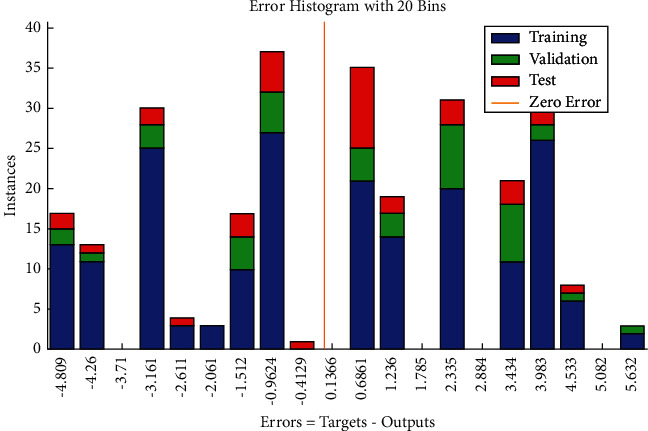
LM error histogram of time-related risks.

The error histogram which shows the distribution of errors for training, validation, and test is shown in Figure 6. The frequency corresponding to positive values of residuals slightly exceeds the frequency corresponding to negative values of errors comparatively.

**Figure 7:**
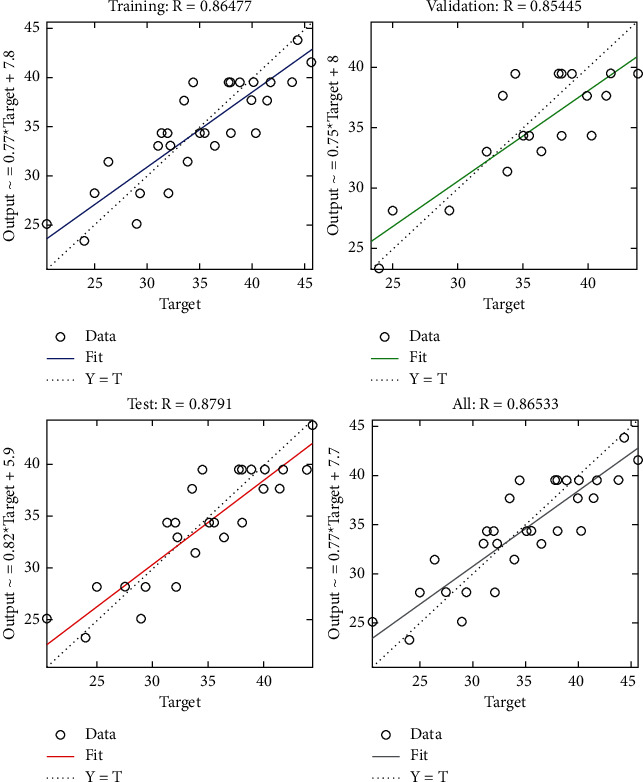
LM regression plot of time-related risks.

The regression plot of time-related risks (see Figure 7) gives a comparison of goodness of fit measure *R* for training, validation, and test and overall taking the target on the horizontal axis and different functional structures on the vertical axis for all four specifications. The values of *R* measure in all four regression plots are not significantly different.

**Figure 8:**
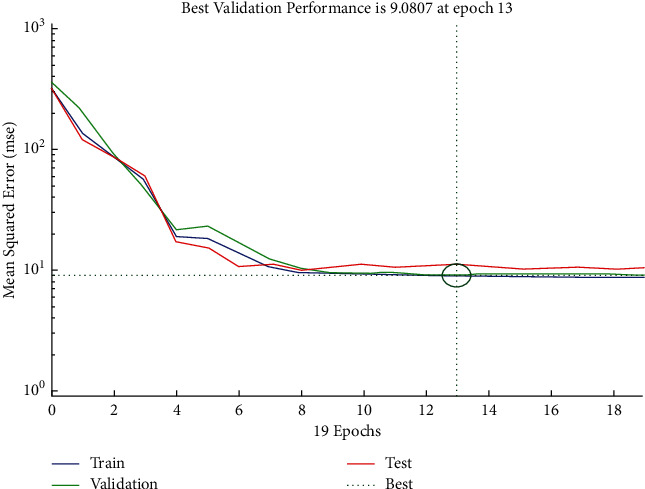
SCG performance plot of time-related risks.

The performance plot of time-related risk in which 19 Epoch values are plotted versus mean squared error using Scaled Conjugate approach is shown in Figure 8. The plot shows the decreasing exponential behavior between 0 and 8 and then follows approximately the horizontal line trend for all values above 8.

**Figure 9:**
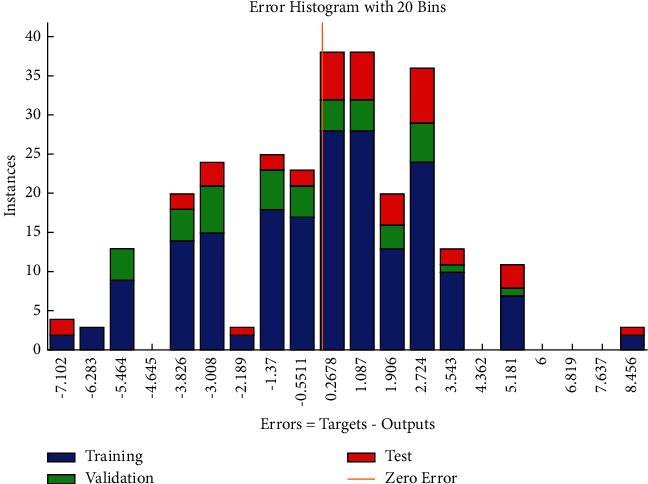
SCG error histogram of time-related risks.

The error histogram (see Figure 9) shows the distribution of errors for training, validation, the frequencies of positive values of residuals, or errors exceeding the frequencies corresponding to negative values.

**Figure 10:**
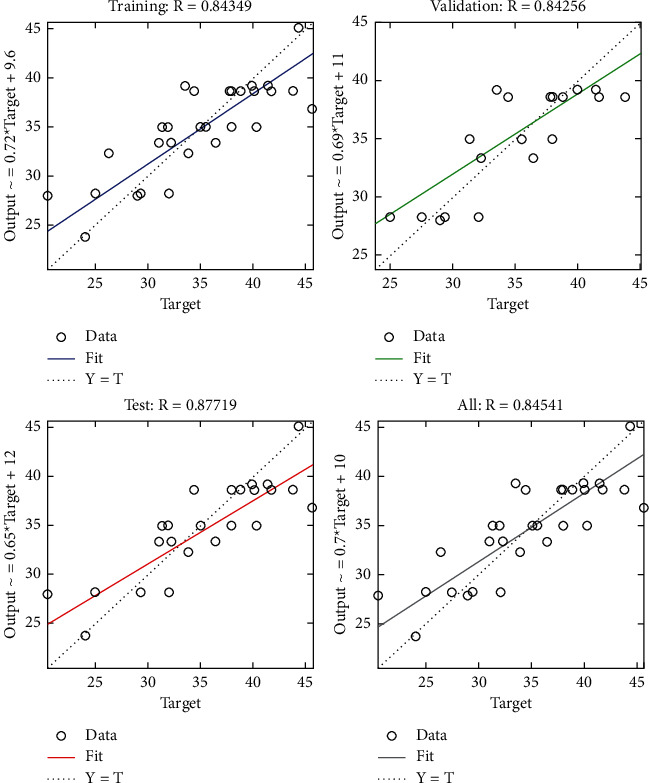
Scaled conjugate regression plot of time-related risks.

The regression plot of time-related risks (see Figure 10) gives a comparison of goodness of fit measure *R* for training, validation, and test and overall taking the target on the horizontal axis and different functional structures on the vertical axis for all four specifications. The values of *R* measure in case of test are significantly greater as compared with those of the other three regression plots.

**Figure 11:**
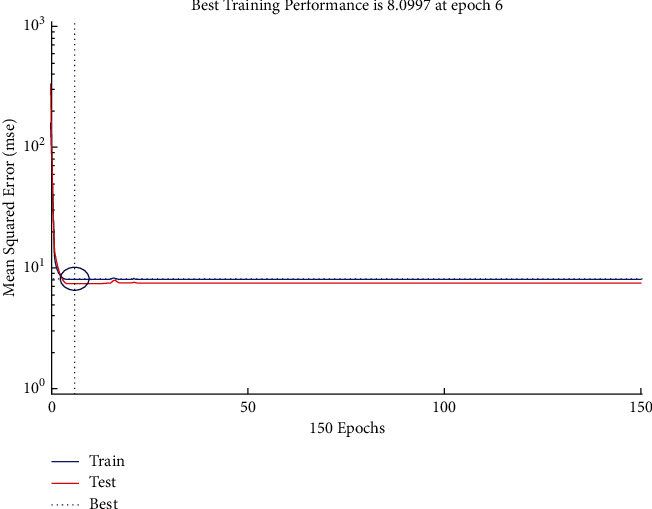
BR performance plot of time-related risks.

The performance plot of time-related risk in which 150 Epoch values are plotted versus mean squared error based on Bayesian approach is shown in Figure 11. The plot shows the *L*-shaped distribution starting from 0 and follows the horizontal line trend for all values above 6.

**Figure 12:**
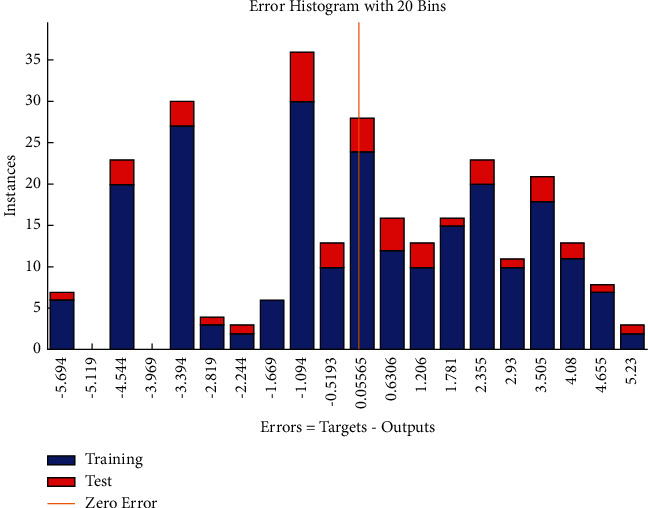
BR error histogram of time-related risks.

The error histogram (see Figure 12) shows the distribution of errors for training, validation, and test for the Bayesian Regularization. The frequencies of negative values of residuals are found more variable as compared with the frequencies corresponding to positive values that follow approximately symmetrical behavior.

**Figure 13:**
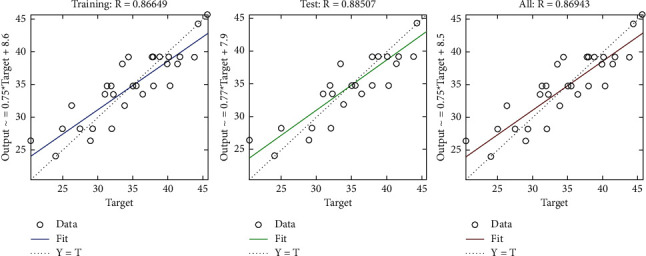
BR regression plot of time-related risks.

The regression plot of time-related risks (see Figure 13) gives a comparison of goodness of fit measure *R* for training, validation, and test and overall taking the target on the horizontal axis and different functional structures on the vertical axis for all four specifications. The values of *R* measure in case of test are relatively greater as compared with those of the other two regression plots.

The results are summarized in Table 5 for cost-related risks taken as the explanatory variable to predict the overall risk associated to global software development. The results reveal that the MSE (validation) has the least value as compared with the other two approaches whereas MSE (testing) value of Scaled Conjugate Gradient is least with the highest value of *R* as compared with the results for the same indicators of the other two approaches and performance has the highest value in case of the Levenberg–Marquardt method. Epoch is computed with the least number of iterations in case of Levenberg–Marquardt as compared with the number of iterations associated to the other two approaches.

**Figure 14:**
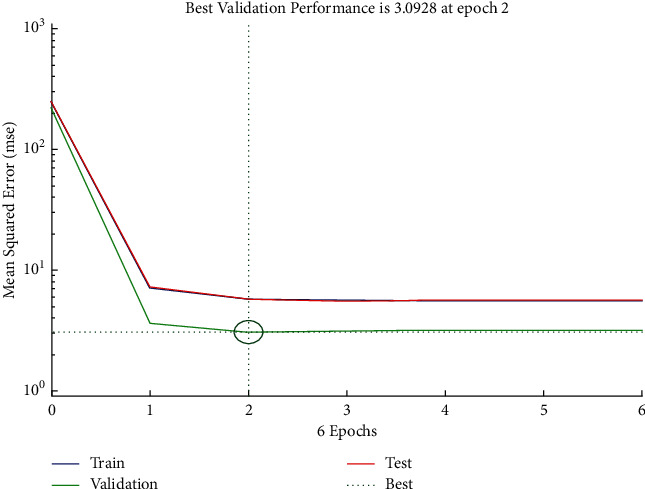
LM performance plot of cost-related risk.

**Figure 15:**
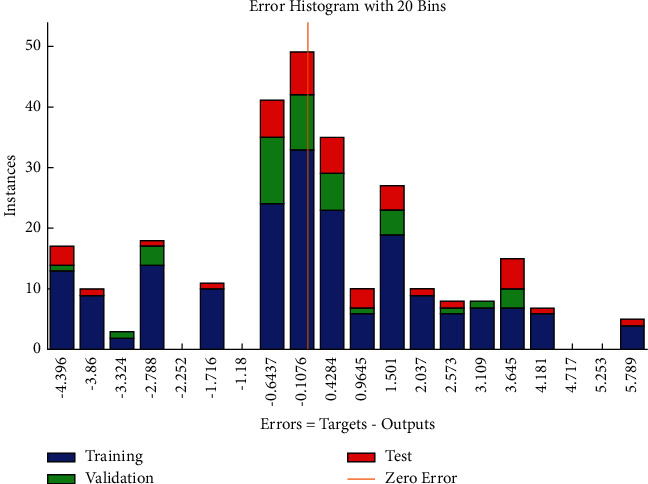
LM error histogram of cost-related risks.

The performance plot of cost-related risk in which 6 Epoch values are plotted versus mean squared error based on Levenberg–Marquardt approach is shown in Figure 14. The plot shows the linearly decreasing behavior from 0 to 1, and it follows approximately the horizontal line trend for all values above 1. It also indicated that the best validation performance is at Epoch 2. The error histogram (see Figure 15) shows the distribution of errors for training, validation, and test.

**Figure 16:**
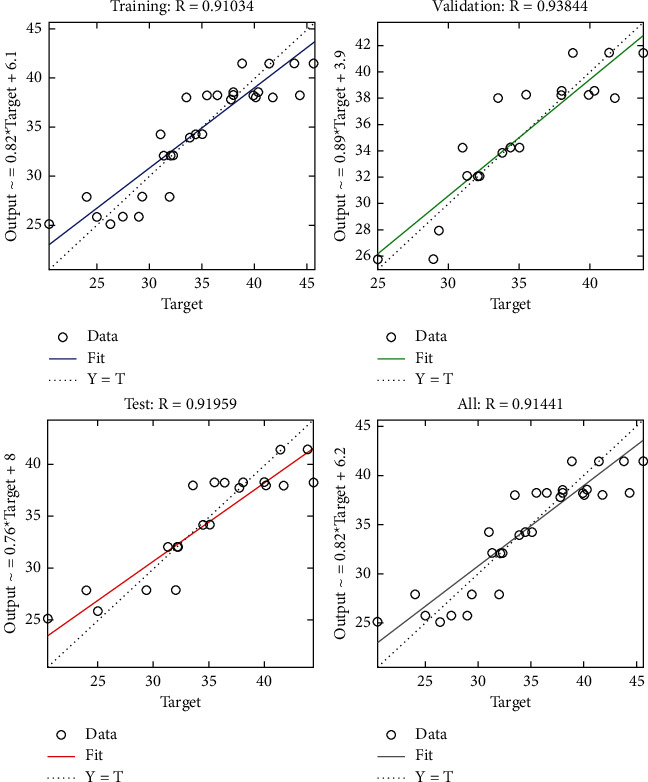
LM regression plot of cost-related risks.

The regression plot of cost-related risks (see Figure 16) gives a comparison of goodness of fit measure *R* for training, validation, and test and overall taking the target on the horizontal axis and different functional structures on the vertical axis for all four specifications. The value of *R* measure of validation is relatively greater as compared with that of the other four regression plots.

**Figure 17:**
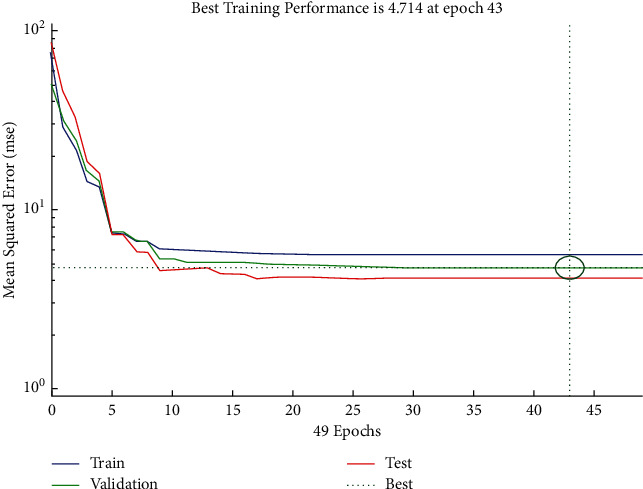
SCG performance plot of cost-related risks.

The performance plot of cost-related risk in which 49 Epoch values are plotted versus mean squared error using Scaled Conjugate approach is shown in Figure 17. The plot shows the best validation performance at Epoch 43.

**Figure 18:**
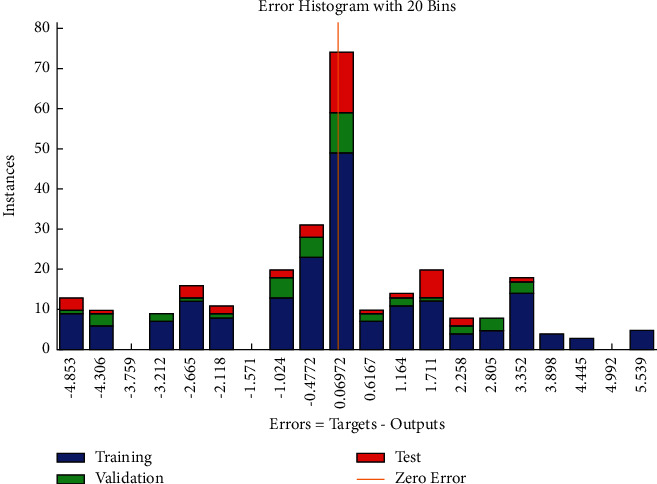
SCG error histogram of cost-related risks.

The error histogram (see Figure 18) shows the approximately symmetrical distribution of errors for training, validation, and test for the Scaled Conjugate approach with slightly greater occurrences of frequencies of negative values of errors as compared with positive values.

**Figure 19:**
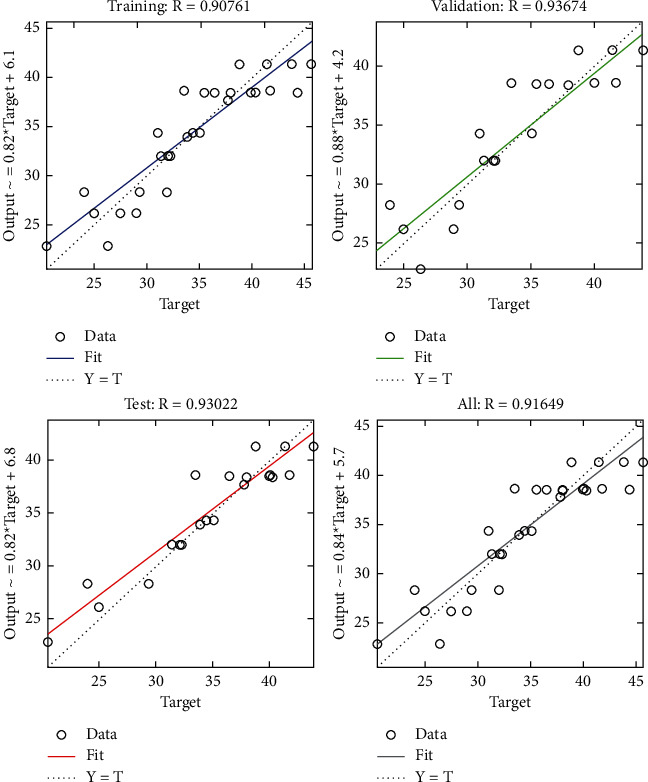
SCG regression plot of cost-related risks.

The regression plot of cost-related risks (see Figure 19) gives a comparison of goodness of fit measure *R* for training, validation, and test and overall taking the target on the horizontal axis and different functional structures on the vertical axis for all four specifications. The values of *R* measure in case of test and validation are nearly same.

**Figure 20:**
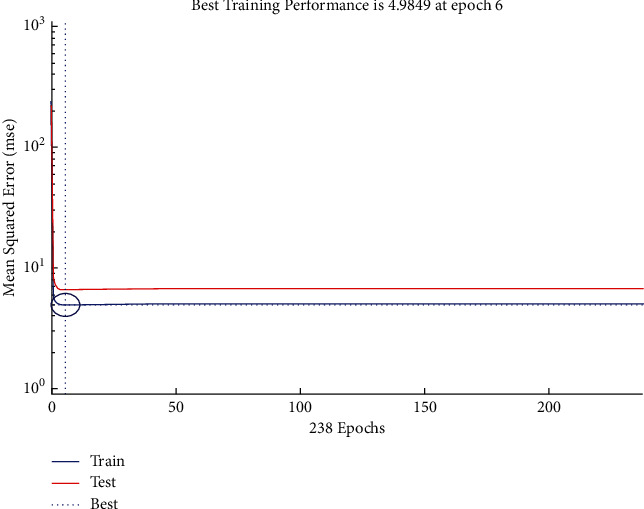
BR performance plot of cost-related risks.

The performance plot of cost-related risk in which 238 Epoch values are plotted versus mean squared error based on Bayesian approach is shown in Figure 20. The plot shows the *L*-shaped distribution starting from 0 and follows the horizontal line trend for all values above 0. It also indicates the best training performance at Epoch 6.

**Figure 21:**
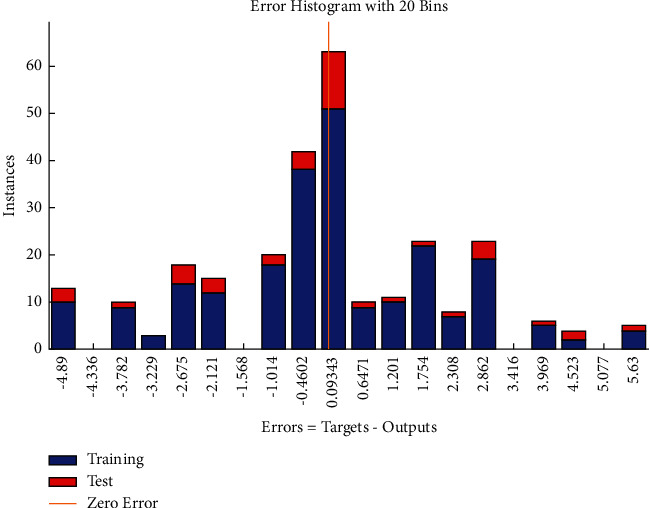
BR error histogram of cost-related risks.

The error histogram (see Figure 21) shows that the frequencies corresponding to negative values of errors are relatively higher as compared with the frequencies corresponding to positive values.

**Figure 22:**
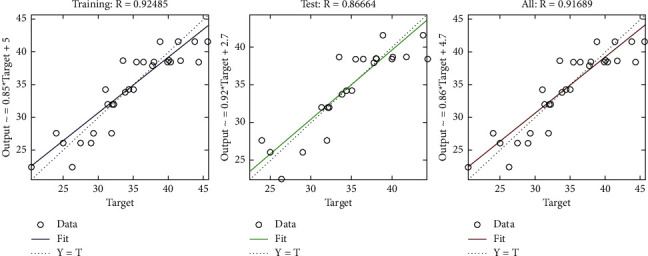
BR regression plot of cost-related risks.

The regression plot of cost-related risks (see Figure 22) gives a comparison of goodness of fit measure *R* for training and test and overall taking the target on the horizontal axis and different functional structures on the vertical axis for all three specifications. It can be observed that the value of *R* measure in case of training is significantly greater as compared with that of test.

The results of the three neural network approaches used to determine the effect of resource-related risks on the overall risk linked with global software development are presented (see Table 6). The results identified that the MSE (validation) of Bayesian Regularization has the least value as compared with the other two alternative approaches used in this study. MSE (testing) value of Levenberg–Marquardt approach is least with the highest value of *R* as compared with the results for the same indicators of the other two approaches whereas performance has the highest value in case of the Levenberg–Marquardt method. Epoch is computed with the least number of iterations in case of Levenberg–Marquardt as compared with the number of iterations associated to the other two approaches.

**Figure 23:**
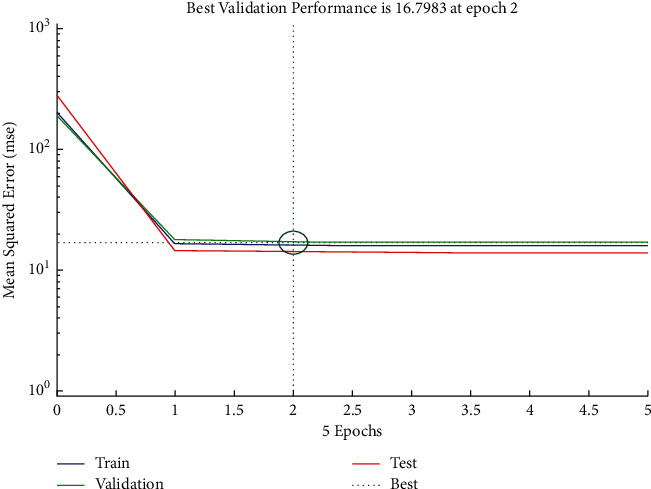
LM performance plot of resource-related risk.

The performance plot of resource-related risk in which 6 Epoch values are plotted versus mean squared error based on LM approach is shown in Figure 23. The plot shows the linearly decreasing behavior from 0 to 1, and then it follows approximately the horizontal line trend for all values above 1. It also indicated that the best validation performance is at Epoch 2.

**Figure 24:**
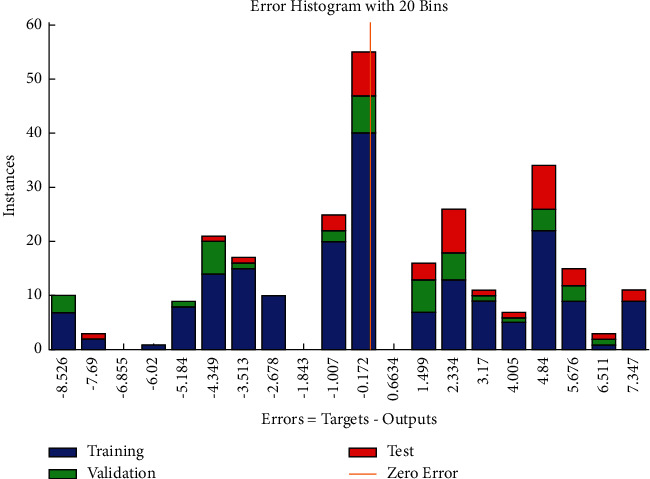
LM error histogram of resource-related risk.

The error histogram (see Figure 24) shows the distribution of errors for training, validation, and test. The negative values of residuals found more variable as compared with the frequencies corresponding to positive values.

**Figure 25:**
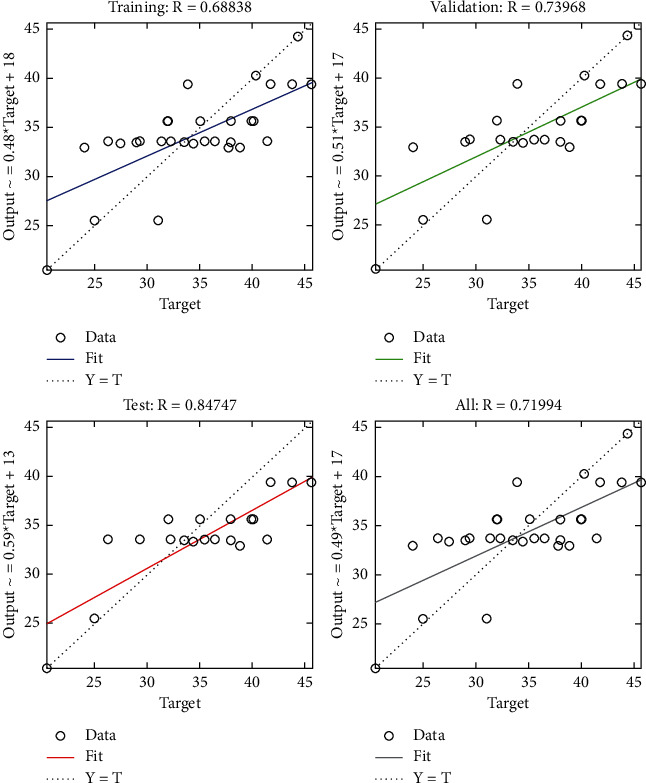
LM regression plot of resource-related risk.

The regression plot of resource-related risks (see Figure 25) gives a comparison of goodness of fit measure *R* for training, validation, and test and overall taking the target on the horizontal axis and different functional structures on the vertical axis for all four specifications. The values of *R* measure for all three plots of training, test, and validation are significantly different which is least in case of training and highest in case of test.

**Figure 26:**
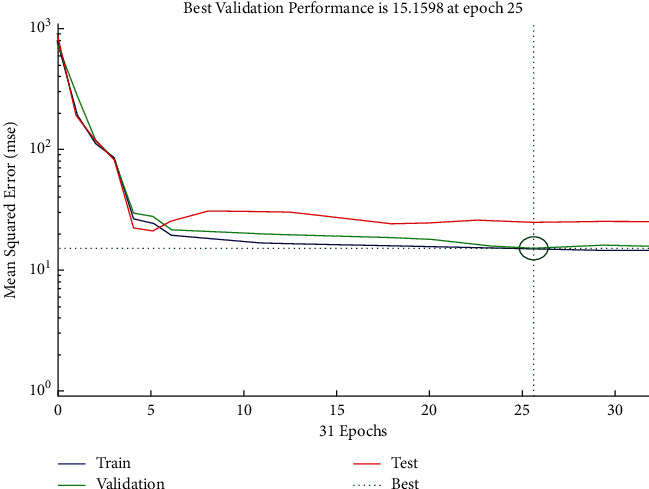
SCG performance plot of resource-related risks.

The performance plot of resource-related risk in which 31 Epoch values are plotted versus mean squared error using Scaled Conjugate approach is shown in Figure 26. The plot shows the decreasing exponential behavior between 0 and 10 and then follows the horizontal line trend, and it gives best validation performance at Epoch 25.

**Figure 27:**
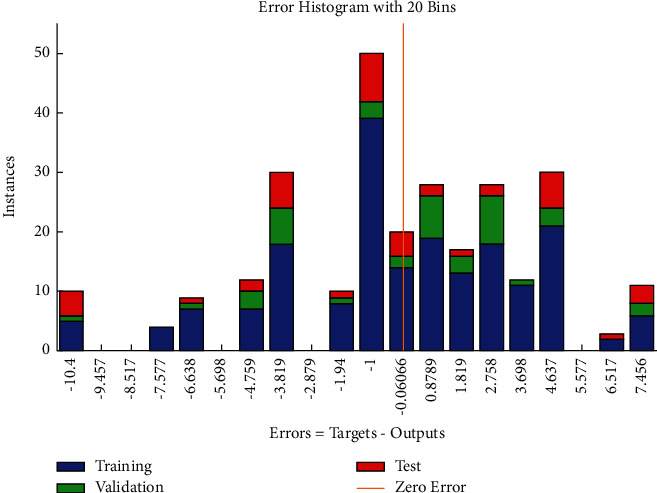
SCG error histogram of resource-related risks.

The error histogram (see Figure 27) exhibits the frequency distribution of errors with greater occurrences for positive values of residuals and relatively low frequencies corresponding to negative values of errors with greater variability.

**Figure 28:**
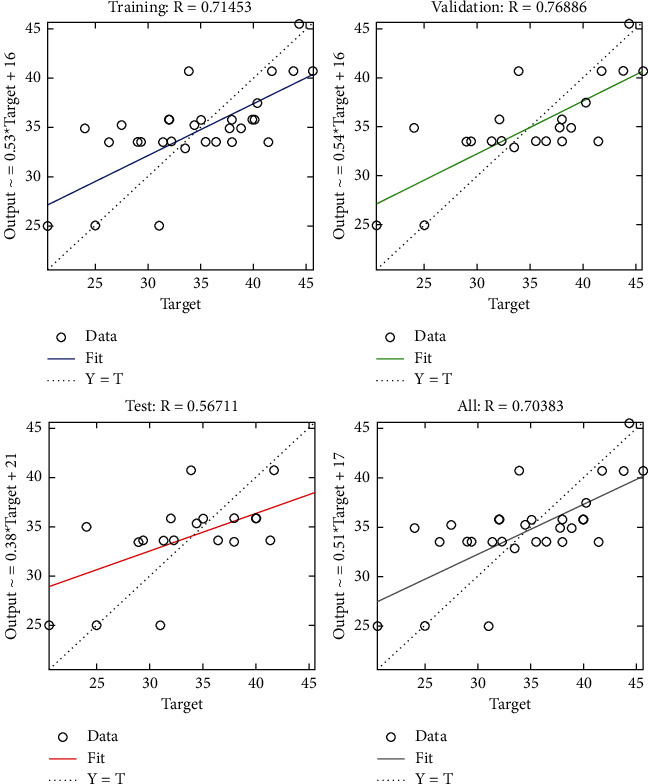
SCG regression plot of resource-related risks.

The regression plot of resource-related risks (see Figure 28) gives a comparison of goodness of fit measure *R* for training, validation, and test which are significantly different in which test has the least value of *R* and validation has the highest value.

**Figure 29:**
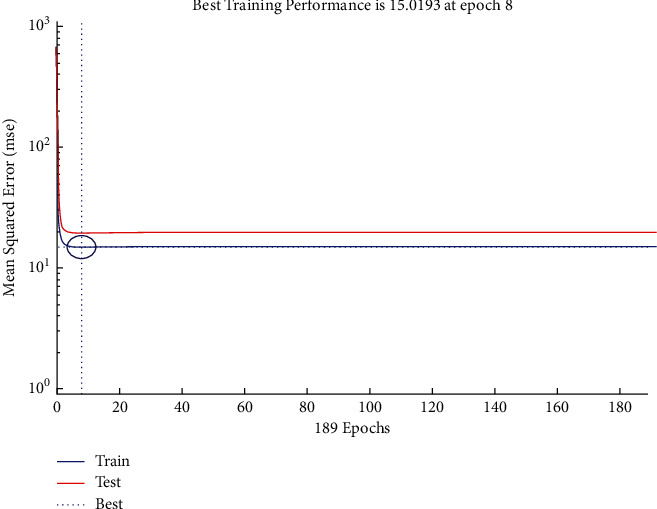
BR performance plot of resource-related risks.

The performance plot of resource-related risk in which Epoch values plotted versus mean squared error based on Bayesian approach indicate the best training performance at Epoch 8 is shown in Figure 29. The plot shows the *L*-shaped distribution starting from 0 and follows the horizontal line trend for all values above 0.

**Figure 30:**
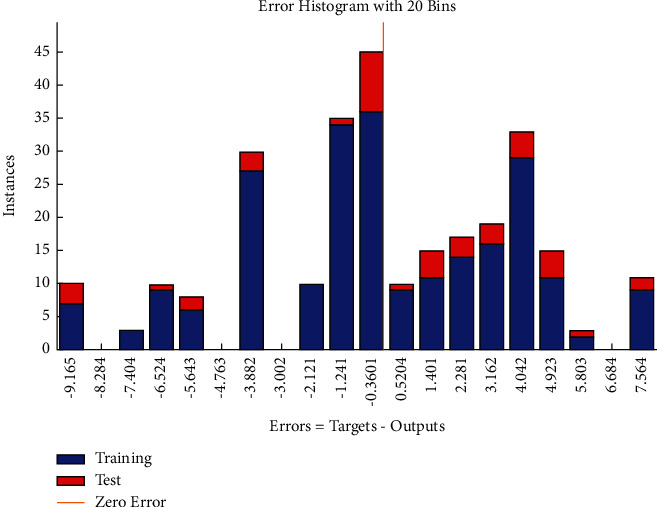
BR error histogram of resource-related risks.

The error histogram (see Figure 30) shows that the frequencies corresponding to negative values of errors are relatively fewer as compared with the frequencies corresponding to positive values.

**Figure 31:**
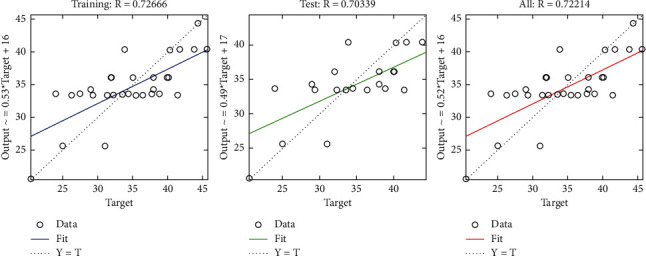
BR regression plot of resource-related risks.

The regression plot of resource-related risks (see Figure 31) gives a comparison of goodness of fit measure *R* for training and test and overall taking the target on the horizontal axis. It can be observed that the value of *R* measure in case of training is significantly greater as compared with that of test.

## 7. Conclusions

The GSD is a complex software development environment in which teams are dispersed and located in different geographical locations and perform their duties in different time zones. Therefore, the risk management pertinent to the software development environment seems difficult. Consequently, there is a need to design and implement proper risk management practices to minimize the impact of risk associated with GSD environment. In this research, neural network training algorithms like Levenberg–Marquardt, Bayesian Regularization, and Scaled Conjugate Gradient have been implemented to predict the responses of risks related to project time, cost, and resource involved in global software development. The data set has been trained using these neural network algorithms, and mean squared rrror (MSE), regression *R* values, processing time, performance, and gradient have been calculated. Comparative analysis of the implemented algorithms has also been performed. The results revealed that the MSE (validation) of Bayesian Regularization in time-related, cost-related, and resource-related risks has the least value as compared with the other two alternative approaches used in this study. Hence, the results proved that Bayesian Regularization gave better results as compared with the other two approaches. This finding is similar to [28, 50] but different from [33].

GSD faces heterogeneous work environments that results in multiple challenges which have been highlighted in this research study. This paper points out the importance of time, cost, and resource risks within GSD. This paper further provides a useful insights of various risk factors within GSD and provided a scalable and generalizable solution to manage them using effective risk prediction. To the best of our knowledge, this is the first study concerned with the risk prediction in the context of GSD using artificial neural network. Three ANN methods are used (Levenberg–Marquardt, Bayesian Regularization, and Scaled Conjugate Gradient) to compare predictive abilities of these methods. The results of this study show that Levenberg is not as efficient as Bayesian regularization and Scaled Conjugate are. After dataset training, the Bayesian Regularization method gave less error in prediction as compared with Levenberg–Marquardt and Scaled Conjugate. Bayesian Regularization provides a robust model in quantitative studies.

Overall findings of this research indicate that all three risks, namely, time, cost, and resource related to the GSD are found significant and critically important for the overall risk associated with GSD. The research findings imply that these factors that are affecting the overall risk must be given due importance and should be minimized to optimize the benefits of GSD. To increase the validity of the findings, this research could also be revisited by including greater span of countries where the operations related to GSD are closely linked. This will help to study the effects of any changes in the nature and intensity in the factors with the greater degree of certainty so that it gives a more reliable framework to optimize the performance of GSD.

It is recommended to use artificial neural network methods, namely, Levenberg–Marquardt, Scaled Conjugate, and Bayesian Regularization for prediction purpose specially on nonlinear data because several researchers have used these three methods with a variety of applications [32–34]. ANN provides a robust model in quantitative studies. The literature in this study proved that these ANN methods outperform other techniques when predicting software-related issues like faults or defects, complexity, and risks [29–31].

### 7.1. Limitations

In this research, the data have been collected from Pakistan, Australia, and the USA. Future study will include other countries to broaden the scope of research. It will be of great help to identify the similarities and dissimilarities regarding the trends related to global software development in different countries of the world. Furthermore, the data are collected on the basis of convenience sampling that prevents the researchers to generalize the results. The use of random sampling will overcome this problem, and the result and findings of the study could be generalized.

The activities related to global software development are the sole responsibility of the team leaders or project managers involved in the survey. Therefore, we receive only one response as only representation from an organization and that is from the project managers' perspective, and consequently it limits the diversity of opinions and perceptions regarding risks related to global software development within an organization.

To identify the risks associated with the global software development, deep learning algorithms may also be used as an alternative to get a greater insight into the subject area as deep learning outperforms machine learning techniques for larger sample sizes. Therefore, the study may be replicated with greater sample size to increase the validity of results.

## Figures and Tables

**Table 1:** List of 26 risks related to time, cost, and resource shortlisted by practitioners.

Risk dimension	Risk no.	Question	Risk factor
Time	R1	Q13	Lack of ineffective PM methodology
	R2	Q14	Inappropriate task timings
	R3	Q15	Failure to provide resources
	R4	Q17	Failure in activity estimation and scheduling
	R5	Q19	Inappropriate planning
	R6	Q20	Unrealistic time estimate
	R7	Q26	Cost overruns
	R8	Q27	Inexperienced project manager
	R9	Q28	Project progress not monitored closely enough

Cost	R10	Q8	Lack of balance on the project team
	R11	Q13	Lack of ineffective PM methodology
	R12	Q14	Inappropriate task timings
	R13	Q15	Failure to provide resources
	R14	Q26	Cost overruns
	R15	Q27	Inexperienced project manager
	R16	Q28	Project progress not monitored closely enough

Resource	R17	Q8	Lack of balance on the project team
	R18	Q10	Inadequately trained development team members
	R19	Q11	Organizational and cultural differences of participants
	R20	Q15	Failure to provide resources
	R21	Q16	Lack of cooperation and coordination among team members
	R22	Q21	Loss of key resource(s) that impact the project
	R23	Q22	Inadequate technical resources
	R24	Q23	Lack of appropriately skilled resources
	R25	Q24	Scope creep
	R26	Q25	Project milestones not clearly defined

**Table 2:** Sample data set Part I (from total of 274 data set).

Country	Q1	Q2	Q3	Q4	Q5	Q6	Q7	Q8	Q9	Q10	Q11	Q12	Q13	Q14	Q15
AUS	1	1	0	1	1	4	2	3	3	3	3	3	4	4	1
AUS	0	0	0	1	1	2	2	3	3	1	1	3	3	1	1
AUS	2	2	1	1	1	1	2	1	3	3	3	1	3	1	1
AUS	1	1	0	1	1	2	2	1	3	3	3	3	3	1	3
AUS	2	1	0	1	1	2	2	3	3	1	1	1	3	1	1
AUS	3	2	0	1	1	3	2	3	3	1	1	3	3	3	1
PAK	2	1	1	1	1	2	2	3	0	3	3	3	3	2	3
PAK	2	2	2	1	1	1	2	2	2	2	1	0	3	3	3
PAK	3	2	1	1	1	1	2	3	1	1	1	0	1	3	1
PAK	0	0	0	1	1	2	2	3	3	1	1	3	3	1	1
PAK	3	2	0	1	1	2	2	2	1	1	1	3	3	3	1
USA	2	1	0	1	1	1	2	4	3	4	4	3	3	1	0
USA	3	1	0	1	1	1	2	3	2	2	2	3	3	3	3
USA	2	1	1	1	1	2	2	1	4	1	1	3	3	3	0
USA	1	1	0	1	1	2	2	1	3	3	3	3	3	1	3
USA	3	2	1	1	1	3	2	3	3	1	1	3	3	3	1
USA	2	1	0	1	1	2	2	3	3	1	1	1	3	1	1

**Table 3:** Sample data set Part II (from total of 274 data set).

Q16	Q17	Q18	Q19	Q20	Q21	Q22	Q23	Q24	Q25	Q26	Q27	Q28	Q29	Q30	Output
3	1	3	4	4	3	3	3	4	4	1	4	4	3	0	3
3	4	3	4	4	3	1	3	4	4	1	4	4	3	0	2
3	3	1	3	3	3	1	3	3	3	1	4	4	1	1	2
3	3	3	4	4	3	1	3	4	4	1	4	3	1	0	0
3	3	3	3	3	3	3	3	4	4	3	3	3	3	0	3
3	1	3	4	4	3	3	3	4	4	1	4	4	3	0	3
4	4	4	3	4	3	3	3	3	4	4	3	3	3	1	3
3	3	3	3	3	3	3	3	3	3	2	3	3	2	0	1
3	3	3	3	3	3	1	2	3	3	3	3	1	2	0	0
4	3	3	4	4	2	3	3	3	3	1	3	3	2	0	1
3	3	1	4	4	3	3	3	3	3	2	3	3	1	1	2
4	3	1	4	4	4	4	3	3	3	2	3	3	2	0	3
2	3	4	4	3	3	3	3	3	3	3	3	2	2	0	2
3	3	3	4	4	3	3	3	3	3	1	3	3	1	1	3
3	3	1	3	3	3	1	3	3	3	1	4	4	1	1	2
3	3	3	3	3	3	3	3	4	4	3	3	3	3	0	3
3	3	3	4	4	3	1	3	4	4	1	4	3	1	0	1

**Table 4:** Comparison of LM, SCG, and BR of time-related risks.

	Levenberg–Marquardt	Scaled Conjugate Gradient	Bayesian Regularization
Epoch	5 iteration	19 iteration	150 iteration
Processing	0 : 00 : 00	0 : 00 : 00	0 : 00 : 02
Performance	8.27	8.71	8.1
Gradient	0	3.16	0.0555
MU	0		5.00 E+10
MSE (validation)	8.02	9.08	2.157
R (validation)	0.85	0.84	0.9245
MSE (testing)	6.90	11.25	7.47
R (testing)	0.88	0.88	0.89

**Table 5:** Comparison of LM, SCG, and BR of cost-related risks.

	Levenberg–Marquardt	Scaled Conjugate Gradient	Bayesian Regularization
Epoch	6 iterations	49 iterations	238 iterations
Processing	0 : 00 : 00	0 : 00 : 00	0 : 00 : 01
Performance	5.63	5.61	5.05
Gradient	0.00	0.0302	0.0516
MU	0.00		5.00e+10
MSE (validation)	3.09	4.71	1.75
R (validation)	0.94	0.94	0.8524
MSE (testing)	5.78	4.14	6.70
R (testing)	0.92	0.93	0.87

**Table 6:** Comparison of LM, SCG, and BR of resource-related risks.

	Levenberg–Marquardt	Scaled Conjugate Gradient	Bayesian Regularization
Epoch	5 iterations	31 iterations	189 iterations
Processing	0 : 00 : 00	0 : 00 : 00	0 : 00 : 01
Performance	16.1	14.6	15.1
Gradient	0.00	3.02	0.124
MU	0.00		5.00e+10
MSE (validation)	16.80	15.16	10.254
R (validation)	0.74	0.77	0.7452
MSE (testing)	14.40	25.04	19.55
R (testing)	0.85	0.57	0.70

## Data Availability

The survey data used to support the findings of this study have not been made available because dataset confidentiality has to be maintained due to PhD studies. However, sample dataset has been incorporated in the article.

## Supplementary Materials

Supplementary Materials The questionnaire developed in this study covers the items related to the project time, cost, and resource risks that contribute to the overall risks of the global software development projects. On the basis of this questionnaire, data collection has been performed. After successful data collection activity, data have been analyzed and got the desired results, which is present in the article.
